# Supplementary material for: Development of antibody-dependent cell cytotoxicity function in HIV-1 antibodies
Source: eLife. 2021 Jan 11;10:e63444. doi: 10.7554/eLife.63444 (PMC7884072; doi:10.7554/eLife.63444)
Supplement: Transparent reporting form [file elife-63444-transrepform.docx]

***eLife’s* transparent reporting form**

We encourage authors to provide detailed information *within their submission* to facilitate the interpretation and replication of experiments. Authors can upload supporting documentation to indicate the use of appropriate reporting guidelines for health-related research (see [EQUATOR Network](http://www.equator-network.org/%20)), life science research (see the [BioSharing Information Resource](https://biosharing.org/)), or the [ARRIVE guidelines](http://www.plosbiology.org/article/info:doi/10.1371/journal.pbio.1000412) for reporting work involving animal research. Where applicable, authors should refer to any relevant reporting standards documents in this form.

If you have any questions, please consult our Journal Policies and/or contact us: [editorial@elifesciences.org](mailto:editorial@elifesciences.org).

**Sample-size estimation**

- You should state whether an appropriate sample size was computed when the study was being designed
- You should state the statistical method of sample size computation and any required assumptions
- If no explicit power analysis was used, you should describe how you decided what sample (replicate) size (number) to use

Please outline where this information can be found within the submission (e.g., sections or figure legends), or explain why this information doesn’t apply to your submission:

Sample-size estimation does not apply to this submission because this study required our use of all available sequencing data generated from unique and limited human blood samples. Sample sizes are not relevant to computational inference of antibody development pathways.

**Replicates**

- You should report how often each experiment was performed
- You should include a definition of biological versus technical replication
- The data obtained should be provided and sufficient information should be provided to indicate the number of independent biological and/or technical replicates
- If you encountered any outliers, you should describe how these were handled
- Criteria for exclusion/inclusion of data should be clearly stated
- High-throughput sequence data should be uploaded before submission, with a private link for reviewers provided (these are available from both GEO and ArrayExpress)

Please outline where this information can be found within the submission (e.g., sections or figure legends), or explain why this information doesn’t apply to your submission:

Technical and biological replicate definitions are in Methods sections “Sequencing of full-length antibody gene variable regions,” “Antibody lineage reconstruction,” and “Epitope mapping with Phage-DMS.”

Technical replicate information is available in the “Replicate” column of Table 1 and in the Methods section “Antibody lineage reconstruction.”

For functional assays (BLI and RFADCC), “independent experiments” are the definition of technical replicates and replicate information is available in each figure legend: Figure 2, Figure 3, Figure 3-Figure supplement 1, Figure 5, Figure 6, Figure 6-figure supplement 1, and Figure 8. For Phage-DMS, legends for Figure 9 and Figure 9-figure supplements 1-2 indicate biological replicate information.

Outliers were not excluded, as there were no statistically-significant outliers in the RFADCC data (GraphPad Prism’s ROUT method with 1% ≤ Q ≤ 10%).

Exclusion criteria are described in Methods sections “Sequence analysis and naïve inference” and “Rapid and fluorometric ADCC (RFADCC) assay”.

Sequencing data are publicly available: BioProject SRA, accessions PRJNA639297 (QA255 antibodies) and PRJNA685289 (Phage-DMS data).

**Statistical reporting**

- Statistical analysis methods should be described and justified
- Raw data should be presented in figures whenever informative to do so (typically when N per group is less than 10)
- For each experiment, you should identify the statistical tests used, exact values of N, definitions of center, methods of multiple test correction, and dispersion and precision measures (e.g., mean, median, SD, SEM, confidence intervals; and, for the major substantive results, a measure of effect size (e.g., Pearson's r, Cohen's d)
- Report exact p-values wherever possible alongside the summary statistics and 95% confidence intervals. These should be reported for all key questions and not only when the p-value is less than 0.05.

Please outline where this information can be found within the submission (e.g., sections or figure legends), or explain why this information doesn’t apply to your submission:

Statistical analyses are described in the Methods section “Quantification and Statistical Analysis.” Information about RFADCC assay positivity thresholds and assay variability can be found in the Methods section “Rapid and fluorometric ADCC (RFADCC) assay.”

(For large datasets, or papers with a very large number of statistical tests, you may upload a single table file with tests, Ns, etc., with reference to sections in the manuscript.)

**Group allocation**

- Indicate how samples were allocated into experimental groups (in the case of clinical studies, please specify allocation to treatment method); if randomization was used, please also state if restricted randomization was applied
- Indicate if masking was used during group allocation, data collection and/or data analysis

Please outline where this information can be found within the submission (e.g., sections or figure legends), or explain why this information doesn’t apply to your submission:

Group allocation does not apply to this submission, as there are no experimental groups. Antibody lineage members (the only “groups” in this submission) were defined computationally, as described in Methods section “Sequence analysis and naïve inference.”

**Additional data files (“source data”)**

- We encourage you to upload relevant additional data files, such as numerical data that are represented as a graph in a figure, or as a summary table
- Where provided, these should be in the most useful format, and they can be uploaded as “Source data” files linked to a main figure or table
- Include model definition files including the full list of parameters used
- Include code used for data analysis (e.g., R, MatLab)
- Avoid stating that data files are “available upon request”

Please indicate the figures or tables for which source data files have been provided:

Figures 2, 3, 5, 6, 8, and 9. For Tables 1 and 2, source data is available as sequencing datasets with BioProject accessions PRJNA639297 and PRJNA685289.
